# Edible Bird’s Nest as a Multi-Component Functional Food for Brain Aging: From Single-Bioactive Actions to Network-Regulatory Mechanisms

**DOI:** 10.3390/nu18040671

**Published:** 2026-02-18

**Authors:** Wenjuan Gong, Xintong Wang, Wen Zhang, Huihui Wang, Wei Xiong, Yixuan Li, Pengcheng Wen, Yanan Sun

**Affiliations:** 1 Key Laboratory of Precision Nutrition and Food Quality, Department of Nutrition and Health, China Agricultural University, Beijing 100083, China; gongwj2023@163.com (W.G.); xtwang@cau.edu.cn (X.W.); zhangwen02919@126.com (W.Z.); whhlyh1227@163.com (H.W.); liyixuan@cau.edu.cn (Y.L.); 2 College of Food Science and Engineering, Gansu Agricultural University, Lanzhou 730070, China; 3 Food Laboratory of Zhongyuan, Luohe 462000, China; xiongwei@zyfoodlab.cn

**Keywords:** functional foods, edible bird’s nest, brain aging, multi-target effects, food-derived sources

## Abstract

Functional food research has long emphasized isolated bioactive compounds, yet such single-target strategies often show limited efficacy against complex, multifactorial processes such as brain aging. In this review, we examine edible bird’s nest (EBN) as a representative multi-component functional food and discuss how its complex food matrix may exert coordinated neuroprotective effects. We summarize the major bioactive constituents of EBN, including sialic acid, functional glycoproteins, and bioactive peptides, and organize their actions into functional modules related to oxidative stress, neuroinflammation, apoptosis, synaptic maintenance, and neurotrophic support. Emerging evidence on the prebiotic potential of EBN and its modulation of the gut–brain axis is also integrated, highlighting interactions between peripheral metabolic regulation and central nervous system function. By comparing EBN with conventional functional ingredients such as vitamins C and E, coenzyme Q10, curcumin, and omega-3 fatty acids, we propose that EBN represents a distinctive “network-regulatory” food system in which nutritional support and pathway modulation are intrinsically linked. Overall, this review provides a conceptual framework for understanding how complex food matrices can be rationally applied to support brain health and reduce age-related neurodegenerative risk.

## 1. Introduction

Complex dietary foods composed of multiple interacting bioactive constituents have attracted increasing attention in functional food research due to their potential to provide coordinated, multi-target physiological regulation beyond isolated compounds. Representative substances such as ginseng, goji berry, and ganoderma epitomize this preventive wisdom [1,2]. Modern science increasingly recognizes certain dietary foods as promising reservoirs of complex bioactive compounds, whose multi-component nature provides a unique basis for addressing multifaceted health challenges. Among these foods, edible bird’s nest (EBN) occupies a distinctive position as an uncommon but established dietary component in several Asian regions [3]. Composed primarily of the salivary secretions of swiftlets, EBN has been consumed for centuries as a nutrient-dense food and is often regarded as a premium functional ingredient [4]. These findings suggest that EBN represents a complex functional food matrix rather than a single bioactive source, providing a scientific basis for exploring its multi-target regulatory potential in health maintenance. The critical distinction lies in the fundamental difference between EBN’s matrix structure and that of botanical supplements rich in phytochemicals, such as ginseng and goji berries. Most plant-derived functional foods consist primarily of small-molecule secondary metabolites [5], whereas EBN’s biological activity stems from its unique protein–glycoprotein–sialic acid core structure [6]. This compositional characteristic indicates a distinct mechanism of action. Therefore, this review proposes a “network regulation” framework that integrates systems biology thinking with food matrix theory. The innovation of this framework lies not in introducing novel biological mechanisms, but in elucidating how naturally complex foods such as EBN achieve coordinated multi-target regulation in the context of brain aging.

The urgency of this research direction stems from the macro-context of global demographic shifts and the specific challenges in brain health. In recent years, the global aging process has accelerated, with projections indicating that by 2050, individuals aged 65 and above will comprise 16% of the global population [7]. This trend is accompanied by a significant increase in the prevalence of age-related neurodegenerative diseases, such as Alzheimer’s disease (AD) and Parkinson’s disease (PD), which pose formidable challenges to global public health systems [8]. Brain aging is a complex pathological process involving multidimensional mechanisms, including oxidative stress, neuroinflammation, mitochondrial dysfunction, protein homeostasis imbalance, and dysregulation of the gut–brain axis [9]. Owing to its high metabolic rate, lipid-rich composition, and relatively limited antioxidant capacity, the brain is especially vulnerable to redox imbalance and oxidative injury during aging [10,11]. Consequently, oxidative stress serves as a central initiator, triggering inflammatory cycles that form a vicious circle with gut microbiota dysbiosis, collectively accelerating neuronal damage and cognitive decline [12]. This intricate interplay of multiple mechanisms often renders traditional single-target intervention strategies limited in efficacy.

In light of this context, the adage “prevention is better than cure” has garnered increasing significance, prompting research efforts aimed at identifying multi-targeted, safe intervention strategies derived from natural dietary resources [13]. It is imperative to note that the compelling preclinical rationale for EBN currently far outpaces the clinical evidence in humans. High-quality randomized controlled trials (RCTs) investigating its effects on cognitive outcomes are notably absent. Therefore, this review reframes EBN as a complex food-derived system, whose long history of dietary consumption has prompted modern investigations into its multi-component, network-regulatory functions relevant to brain aging. By organizing EBN bioactive components into functional modules and integrating direct neuroprotective actions with emerging evidence on gut–brain axis modulation, this review seeks to clarify how complex dietary matrices can exert coordinated effects on brain aging. Through comparison with conventional functional ingredients, we further highlight the distinctive advantages of such complex food systems and provide a conceptual framework for their rational application in brain health maintenance and neurodegenerative disease prevention.

## 2. Nutritional Components and Bioactive Substances in EBN

**Figure 1:**
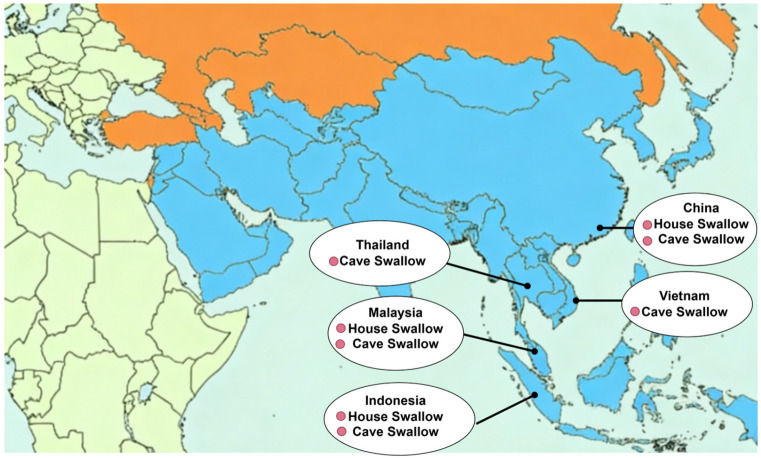
Primary production areas and types of edible bird’s nest (EBN). Blue-shaded regions indicate the major EBN-producing countries in Southeast Asia. Red dots denote representative production types, including house swallow nests and cave swallow nests. The map was created using Figdraw (Version 2.0, www.figdraw.com).

EBN is a nest constructed by swiftlets using their saliva or a mixture of saliva and down feathers. It is primarily produced in Southeast Asian countries such as Indonesia, Malaysia, Thailand, and Vietnam (Figure 1) [4].

**Figure 2:**
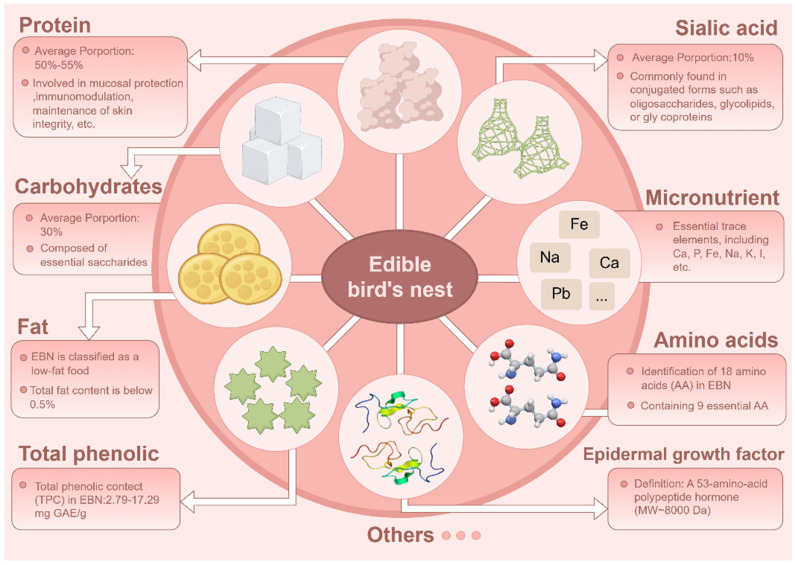
Bioactive nutritional composition of edible bird’s nest (EBN). The primary nutritional constituents of EBN are protein, carbohydrates, sialic acid, amino acids, fats, trace elements, epidermal growth factor (EGF), and total phenols. This figure was created using Figdraw (Version 2.0, www.figdraw.com).

Numerous researchers have investigated the active constituents of EBN (Figure 2), identifying that its nutritional composition predominantly consists of proteins, carbohydrates, amino acids, minor lipids, and trace elements, including calcium (Ca), iron (Fe), and selenium (Se) [14]. The composition of EBN is subject to variation due to factors such as the species of swiftlet, available food resources, nesting season, and processing methods [15]. For example, Quek et al. conducted an analysis of the nutritional composition, physicochemical properties, and antioxidant characteristics of EBN sourced from domesticated swiftlets, brown swiftlets, and the Malay Peninsula. This study considered three dimensions: production methods, swiftlet species, and geographical origin. The total amino acid content in nests of the brown swiftlet (*Aerodramus fuciphagus*) was found to be 64.57 g/100 g, surpassing that of the giant swiftlet (*Aerodramus maximus*) nests by 23%. EBN obtained from farmed brown swiftlets and the Malay Peninsula demonstrated the highest antioxidant activity, ranging from 2.33 to 3.49 mg ascorbic acid equivalent per gram (mg AAE/g), and the highest sialic acid content, measured at 13.57 g/100 g. In contrast, EBN derived from cave-dwelling giant swiftlets and regions in East Malaysia was notably rich in minerals such as calcium and magnesium. The radical scavenging activity of 1,1-diphenyl-2-picrylhydrazyl (DPPH) and the ferrous reduction antioxidant capacity (FRAP) of EBN derived from domesticated brown swiftlets in the Malay Peninsula region are approximately twice as elevated as those from other geographic regions [16].

### 2.1. Proteins

Proteins represent the predominant component of EBN, with an average content ranging from 50% to 55% [17]. Zukefli utilized ultrasonication-assisted methods, detergent-assisted techniques (specifically Triton X-100 and SDS), and Tris-HCl buffer dissolution to analyze and compare the protein profiles of EBN sourced from two distinct Malaysian locations. The results indicated a significantly higher concentration of water-soluble proteins in EBN [18]. Through the application of various separation and analytical techniques, including gel permeation chromatography (GPC) and reverse-phase high-performance liquid chromatography (RP-HPLC), numerous researchers have identified 58 unique protein molecules within EBN. Notable among these are acidic mammalian chitinase (AM Case), lysyl oxidase homologue 3 (LOXL3), mucin-5AC (MUC5AC), ovoinhibitor, Nucleobindin-2 (NUCB2), calcium-binding protein and glucose regulator protein. These proteins are implicated in a range of biological functions, including immunoregulation, mucosal protection, maintenance of skin integrity, and regulation of enzymatic activity [19]. Wang et al. conducted an analysis of the protein composition of four EBN strains sourced from three major production regions using label-free quantitative proteomics, identifying a total of 37 functional proteins. Among these, six core proteins were identified, specifically mucin 5B (MUC5B), lysyl oxidase-like protein 2 (LOXL2), chitinase (CHIA), ovomucoid-like protein (OIH), and calcium-binding protein 45 (CAB45). Gene Ontology (GO) functional annotation revealed that the majority of EBN proteins are classified within the secreted protein category and exhibit various enzymatic activities [20]. Notably, EBN also contains a 66 kDa protein that functions as an IgE-mediated allergen, which has the potential to induce anaphylactic shock in children [21]. Additionally, Yew et al. identified 29 proteins in EBN extract through de novo peptide sequencing utilizing tandem mass spectrometry. These proteins are implicated in various biological mechanisms, including immunoregulation, extracellular matrix formation, neurodevelopment, cell survival and apoptosis, cell proliferation and migration, antioxidant activity, and other fundamental cellular processes. For example, the study demonstrated that EBN contains a variety of proteins that play significant roles in cell proliferation and migration. These include members of the repulsive guidance molecule domain family B, which facilitate neurite extension and axonal growth, as well as proteins such as lin-9 and the hyaluronan-mediated motility receptor, which are also implicated in cell proliferation and migration [22]. Furthermore, bioactive peptides produced through the enzymatic hydrolysis of EBN exhibit a wide range of biological and nutritional properties. Antioxidant peptides derived from this process possess advantageous pharmacokinetic and pharmacodynamic attributes, including high bioavailability in humans, strong antioxidant activity, and minimal adverse effects [14]. For instance, Zhang et al. conducted an extraction of EBN samples using hot water at 100 °C, followed by enzymatic digestion with pepsin (10,000 units), trypsin (36 mg), and bile extract (112.5 mg). The study employed pH variations to mimic the intestinal environment, ranging from 8.9 to 2 and returning to 8.9, in conjunction with a 10 kDa dialysis membrane to simulate gastrointestinal digestion and absorption processes. The results indicated that undigested aqueous extracts of EBN displayed minimal antioxidant activity, whereas digested samples showed a significant enhancement in antioxidant activity at comparable concentrations [23]. In a separate investigation, researchers employed pepsin–trypsin hydrolysis on EBN and identified two novel antioxidant pentapeptides in the resulting hydrolysate: Pro-Phe-His-Pro-Tyr and Leu-Leu-Gly-Asp-Pro. These peptides demonstrated resistance to gastrointestinal proteases and exhibited no cytotoxic effects in vitro on human lung MRC-5 cells, while also providing protection to human hepatocellular carcinoma HepG2 cells against hydroxyl radical-induced damage [24]. Additionally, Lee et al. utilized alkaline enzyme hydrolysis on EBN protein, followed by isolation, purification, and identification of antioxidant and anti-inflammatory peptides using ultrafiltration, gel chromatography, and mass spectrometry. Molecular docking analysis revealed that peptide segment 1 (LFWSPSVYLK) and peptide segment 2 (GWPHLEDNYLDW) possess hydrophobic and antioxidant amino acids, which effectively inhibit the formation of the Keap1-Nrf2 complex through competitive interactions. In contrast, peptide 3 (NPPADLHK) and peptide 4 (GDLAYLDQGHR) demonstrated significant regulatory effects on the IKK-β binding site, facilitated by interactions between their anti-inflammatory amino acids and C-terminal arginine/lysine residues [25].

### 2.2. Carbohydrates

Carbohydrates represent the second most abundant class of substances after proteins, comprising approximately 30% of the total composition. They predominantly consist of seven of the eight essential sugars necessary for human biological activity, including 7.2% N-acetylgalactosamine, 5.3% N-acetylglucosamine, 16.9% galactose, 0.7% fucose, and N-acetylneuraminic acid (sialic acid) [6,26]. The carbohydrates in EBN combine with proteins to form glycoproteins, a complex structure that endows bird’s nest with unique biological activity [27].

### 2.3. Sialic Acids

EBN is a rich dietary source of sialic acid (SA), accounting for approximately 10% of the total, and is typically found in bound forms such as oligosaccharides, glycolipids, or glycoproteins. SA are classified into four distinct types based on the substituent group attached to the carbon 5 position: N-acetylneuraminic acid (Neu5Ac or NANA), N-glycolylneuraminic acid (Neu5Gc), diamino neuraminic acid (KDN), and neuraminic acid (Neu) [28]. In the context of EBN, SA specifically refers to Neu5Ac. This compound is a critical component of EBN, essential for infant brain development as it significantly contributes to synaptic connectivity, neuronal growth, memory formation, and immune system enhancement [29]. Empirical evidence suggests a strong correlation between mammalian cognitive capabilities and variations in brain SA concentrations. Exogenously administered SA is localized within synaptic regions, where it affects the dynamics of positive neurotransmitter movement, neurotransmitter release, and modifications to existing synaptic structures [30]. Furthermore, research indicates that a reduction in both endogenous and exogenous brain SA concentrations can lead to irreversible cognitive decline, although SA supplementation has been shown to enhance learning processes [31].

### 2.4. Amino Acids

Eighteen amino acids have been identified in EBN, including the nine essential amino acids necessary for human growth and repair [32]. Studies suggest that the total essential amino acid content in EBN is 17.8 g/100 g, which is substantially higher than that found in common high protein foods, such as eggs (4.7–7.0 g/100 g) and milk (1.1 g/100 g) [16]. Notably, lysine and tryptophan, which are typically absent in most plant proteins, are present in EBN, rendering it a complete amino acid source for vegetarians. Arginine and serine are the predominant essential and non-essential amino acids, comprising 4.0–4.7% and 4.0–5.5% of the total amino acid content, respectively [33]. Additionally, EBN is enriched with antioxidant amino acids, including proline, histidine, phenylalanine, and tryptophan, indicating its potential utility as a high-quality essential amino acid supplement in dietary nutritional interventions [34].

### 2.5. Fats

EBN is classified as a low-fat food product, characterized by a total fat content of less than 0.5%. The triglyceride profile of EBN demonstrates unique functional properties: polyunsaturated fatty acids represent the largest fraction at 48.43%, with linoleic acid, an essential fatty acid, comprising 47.15% of this category. Saturated fatty acids make up 25.35% of the total, with palmitic acid being the most prevalent at 21.33%. Monounsaturated fatty acids account for 24.74%, primarily consisting of oleic acid at 21.97% [35].

### 2.6. Microelements

EBN is also a source of essential trace elements, including Ca, phosphorus (P), Fe, sodium (Na), potassium (K), and iodine (I). The concentrations of these minerals and metal ions may originate from the swiftlets, the birds responsible for constructing EBN, or from environmental seepage. Due to the diverse geographic origins and types of samples, there is significant variability in their concentration ranges [36]. Studies have reported relatively high Na content (6017 mg/kg), along with notable levels of magnesium (Mg) (344 mg/kg), K (138 mg/kg), and Ca (68 mg/kg). Other minerals such as P, Fe, chromium (Cr), and selenium (Se) are present at concentrations of 0.037, 4.52, 0.30, and 0.14 mg/kg, respectively [15]. However, EBN also contains toxic elements, including lead (Pb), cadmium (Cd), arsenic (As), and mercury (Hg), which necessitates rigorous monitoring of the processing procedures [36].

### 2.7. Epidermal Growth Factor

Epidermal Growth Factor (EGF) is a low molecular weight polypeptide hormone consisting of 53 amino acids, with an approximate molecular weight of 8000 Da. It is integral to cellular and tissue growth, proliferation, differentiation, and development [37,38]. Additionally, EGF significantly influences physiological processes such as cellular damage repair, wound healing, tissue regeneration, and inflammatory responses [34]. In vivo, EGF modulates the proliferation, migration, and neurogenesis of neural stem cells (NSCs). Consequently, it is postulated that EBN extract may exhibit EGF-like activity, producing similar effects on NSCs [22].

### 2.8. Total Phenols

The total phenolic content (TPC) in EBN ranges from 2.79 to 17.29 mg GAE/g, which is higher than that found in honey and vegetables such as broccoli, carrot, and ginger, which have TPC ranges of 1.1–2.0 mg GAE/g and 0.3–4.0 mg GAE/g, respectively [16]. It is important to note that these phenolic compounds, likely derived from the swiftlet’s insectivorous diet or as metabolic byproducts, represent a heterogeneous and minimally characterized fraction within EBN. While contributing to its overall antioxidant capacity, they are not considered the principal bioactive drivers of EBN’s neuroprotective effects, which are more closely linked to its unique proteinaceous and sialylated components (e.g., glycoproteins, SA). Their role may be ancillary, potentially engaging in synergistic interactions within EBN’s complex food matrix.

## 3. The Mechanisms of Brain Aging

**Figure 3:**
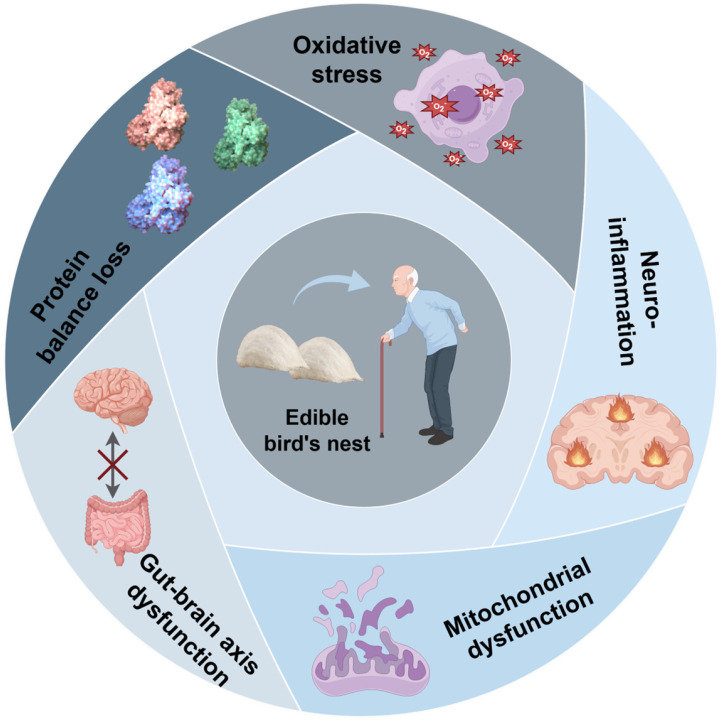
Schematic illustration of the multi-target regulatory mechanisms of edible bird‘s nest (EBN) in counteracting brain aging. EBN is positioned at the center, surrounded by five key pathological processes associated with brain aging: oxidative stress, neuroinflammation, mitochondrial dysfunction, protein balance loss, and gut-brain axis dysfunction. The colored circular icons represent each distinct pathological mechanism. This figure was created using Figdraw (Version 2.0, www.figdraw.com).

Cognitive aging is a multifaceted process characterized by changes in various molecular and cellular pathways, which ultimately contribute to the onset of mild cognitive impairment (MCI), AD, and other dementias [39]. Current widely recognized mechanisms include oxidative stress [40], neuroinflammation [41], mitochondrial dysfunction [42], protein homeostasis imbalance [43], and dysregulation of the gut–brain axis (Figure 3) [44].

### 3.1. Oxidative Stress

Oxidative stress is defined as an imbalance between oxidants and antioxidants in the body, resulting in the excessive production of reactive oxygen species (ROS). These ROS can inflict damage on biomolecules such as DNA, proteins, and lipids [45]. Within neurons, types of ROS include superoxide anions generated by the respiratory chain and various oxidases, hydroxyl radicals formed through the reaction of hydrogen peroxide with copper or ferrous iron, and nitric oxide (NO) produced in response to elevated intracellular Ca^2+^ levels [46]. As individuals age, there is a notable increase in ROS production within neurons, coupled with the accumulation of oxidative damage, which significantly correlates with the severity of neurodegenerative diseases [47]. Morales-Martínez et al. demonstrated, using α-synuclein transgenic rats, a positive correlation between the levels of various oxidative stress biomarkers and neurodegeneration across multiple brain regions [30]. Moreover, ROS generated through oxidative stress and the subsequent cellular damage, such as compromised proteins and lipids, function as ‘danger signals’ that activate microglia and astrocytes, thereby triggering neuroinflammatory responses [48].

### 3.2. Neuroinflammation

Neuroinflammation is characterized by the activation of immune responses by microglia and astrocytes within the central nervous system [49]. Under normal physiological conditions, astrocytes and microglia are essential for maintaining neuronal homeostasis and facilitating waste clearance in the brain [50]. However, when these cells become overactivated, they release substantial quantities of pro-inflammatory cytokines, including IL-1β, IL-6, and TNF-α, as well as chemokines and additional reactive ROS. Moreover, activated microglia express inducible nitric oxide synthase (iNOS), resulting in the production of substantial amounts of NO, which can induce oxidative damage to neurons, culminating in apoptosis and the loss of neuronal function [42]. Studies suggest that with advancing age, microglial migration and phagocytic activity decline, while the population of inflammatory microglia increases by two-to four fold in the brains of aged mice [41]. Additionally, during neuroinflammation, astrocytes upregulate the expression of interleukin-17 (IL-17) and the TrkB transmembrane receptor. IL-17, upon binding to its receptor, may recruit NF-κB activator 1, thereby promoting the production of pro-inflammatory cytokines. During neuroinflammatory conditions, astrocytes may also compromise blood–brain barrier integrity, potentially facilitating the entry of undesirable immune cells and neurotoxic substances into the brain parenchyma [51]. Evidence indicates that the activation of microglia and astrocytes contributes to the aggregation of β-amyloid (Aβ) and the hyperphosphorylation of the microtubule-associated protein Tau within the brain, processes that subsequently precipitate neuronal degeneration and ultimately lead to the onset of AD [52].

Neuroinflammation and oxidative stress exhibit a reciprocal and interconnected relationship, wherein the inflammatory response precipitates oxidative stress, and oxidative stress subsequently activates multiple signaling pathways, including NF-κB, to enhance pro-inflammatory gene expression, thereby intensifying neuroinflammation [53].

### 3.3. Mitochondrial Dysfunction

Mitochondria, essential intracellular organelles, predominantly generate ROS at respiratory chain complexes I and III. These ROS can inflict oxidative damage on mitochondrial DNA (mt DNA), resulting in mt DNA mutations that lead to defective respiratory chain components. This defect, in turn, generates additional ROS, establishing a self-perpetuating cycle of ROS production and mt DNA mutation accumulation [54]. Neurons, due to their intricate morphology and substantial metabolic demands, are particularly reliant on mitochondria and are thus especially vulnerable to mitochondrial dysfunction. As age progresses, mitochondrial capacity to produce and sustain the high energy levels requisite for neuronal function diminishes. When mitochondrial dysfunction surpasses a critical threshold, the brain’s resilience to damage deteriorates, initiating various neurodegenerative processes [55]. Research has identified a detrimental cycle characterized by excessive production of ROS, inflammatory responses, and mitochondrial dysfunction. Mitochondrial damage leads to the activation of glial cells, and the inflammatory mediators produced as a result further impair mitochondrial function, thereby exacerbating neurodegeneration [56]. For example, mitochondrial dysfunction and disrupted mitochondrial autophagy may contribute to amyloid pathology and neurodegeneration in AD [57]. Similarly, mitochondrial dysfunction is a critical hallmark of PD progression, with various risk factors associated with Parkinson’s pathogenesis exerting their effects by inducing mitochondrial dysfunction. In mouse models of PD induced by rotenone, a significant reduction in the activity of mitochondrial complex I has been observed within the striatum [58].

### 3.4. Proteostasis Imbalance

Proteostasis refers to the homeostatic regulation of cellular processes involved in protein synthesis, folding, repair, degradation, and renewal [59]. As organisms age, proteostasis gradually declines, resulting in the accumulation of proteins that are misfolded, oxidized, glycated, or ubiquitinated. This deterioration leads to the formation of toxic protein aggregates [60]. The accumulation of misfolded and aggregated proteins particularly impacts post-mitotic cell types, such as neurons [61]. Additionally, pathological protein aggregation is a prominent hallmark of neurodegenerative diseases. In various neurodegenerative disorders, misfolded proteins abnormally accumulate both intracellularly and extracellularly, forming characteristic aggregates. For example, Aβ and tau protein aggregates are found in AD, alpha-synuclein aggregates in PD, and TAR DNA-binding protein-43 (TDP-43) and Superoxide dismutase 1 (SOD1) aggregates in amyotrophic lateral sclerosis. These protein aggregates exhibit propagating properties, enabling them to spread between neurons [62]. Moreover, protein degradation plays a critical role in memory processes during both normal aging and pathological conditions. Recent studies suggest that hippocampal autophagy is essential for enhancing learning and memory by facilitating activity dependent structural and functional synaptic plasticity. Activation of hippocampal autophagy has been shown to ameliorate age-related memory impairments in murine models. In models of AD in mice, enhancing autophagy has been demonstrated to decrease Aβ accumulation in the brain, thereby restoring synaptic integrity and memory function [63].

### 3.5. Dysfunction of the Gut–Brain Axis

A bidirectional communication system, known as the microbiota–gut–brain axis (MGBA), exists between the central nervous system, gut microbiota, and the gastrointestinal tract [64]. Gut microbiota are pivotal in the processes of aging and the pathogenesis of neurodegenerative diseases, although the intricate signaling pathways involved are not yet fully understood. Currently identified pathways include metabolic, immune, neural, and endocrine signaling mechanisms [65]. In comparison to younger individuals, the gut microbiota of elderly populations undergoes significant alterations. Studies comparing the microbiomes of young and aged rats reveal a notable reduction in microbial diversity in the gut microbiota of older rats, along with distinct compositional differences. For example, in aged rats, there is a decrease in the relative abundance of Bacteroides in the intestines, whereas the abundance of Firmicutes is significantly higher compared to young rats [66]. Simultaneously, the integrity and function of the intestinal barrier decline with age, negatively impacting blood–brain barrier (BBB) permeability and exacerbating neuroinflammation and functional deterioration within the central nervous system [67]. Supporting evidence from further research indicates that during aging, increased intestinal permeability, mediated by the gut microbiota, facilitates the translocation of harmful intestinal metabolites, such as N6-carboxymethyllysine, across the intestinal barrier into the systemic circulation. The accumulation of these metabolites in the brain can lead to microglial damage [68]. Conversely, microbial metabolites, including short-chain fatty acids (SCFAs), tryptophan, cytokines, and neuroactive substances are released into the bloodstream. Once in circulation, the microbiome and its metabolites can modulate peripheral immune cells, interact with the BBB to alter its integrity, and cross the BBB to influence cerebral function [69].

## 4. The Role of EBN Intervention in Brain Aging

Current research provides systematic evidence from both animal models and cellular studies, suggesting that EBN exerts a multi-target protective effect in the context of aging and neurodegenerative diseases (Table 1). In animal studies, EBN has been shown to alleviate neurofunctional impairments induced by various etiological factors through multiple mechanisms. Specifically, in a model of menopause-associated cognitive decline, dietary supplementation with EBN significantly mitigates memory and cognitive deterioration by upregulating the expression of Sirtuin-1 (SIRT1) protein in the hippocampus, downregulating genes associated with neurodegeneration and apoptosis, and modulating redox balance [70,71]. Furthermore, in lipopolysaccharide (LPS)-induced neuroinflammatory models involving Wistar rats and C57BL/6J mice, administration of EBN water extract or EBN itself effectively suppresses abnormal elevations of pro-inflammatory cytokines (such as TNF-α and IL-6) and oxidative stress markers (including MDA), demonstrating anti-inflammatory effects by inhibiting TICAM1 expression within the NF-κB signaling pathway [72,73]. In a mouse model of PD, treatment with specific EBN extracts (pancreatic enzyme-digested extract and aqueous extract) not only enhanced the activity of endogenous antioxidant enzymes, including Superoxide dismutase (SOD) and glutathione peroxidase (GPx), but also inhibited the abnormal activation of microglia, excessive NO production, and lipid peroxidation reactions. These actions collectively contributed to the protection of dopaminergic neurons in the substantia nigra [74]. In models of AD induced by chronic cerebral hypoperfusion, EBN intervention was associated with reduced neuronal loss in the hippocampus and cortex and reduced levels of the oxidative stress marker F2-isoprostane [75]. Additionally, EBN water extract powder demonstrates preventive potential against vascular dementia by inhibiting acetylcholinesterase (AChE) activity, thereby reducing Aβ accumulation [17]. At the cellular level, the aqueous extract of EBN and its functional protein components, lactoferrin and ovotransferrin, exhibited significant antioxidant and anti-apoptotic properties in models of SH-SY5Y cell damage induced by 6-hydroxydopamine (6-OHDA) and hydrogen peroxide [74,76,77]. Comparative studies using different EBN extracts (e.g., pancreatic enzyme-digested vs. aqueous) at maximum non-toxic dose (MNTD) further elucidated their respective efficacies in enhancing cell viability and mitigating oxidative stress and apoptosis [76]. Notably, the specific EBN extracts (crude extract S1 and aqueous extract S2) promoted proliferation, migration, and neuronal differentiation in embryonic mouse neural stem cells (NE-4C), indicating neurotrophic activity [22]. Further investigations reveal that the SA component present in EBN substantially enhances mitochondrial function in SH-SY5Y cells [78]. Importantly, the primary active component, SA, when administered in purified form, exhibits significant independent neuroprotective properties. In double transgenic AD (2 × Tg-AD) mice, SA administration not only enhanced spatial learning and memory abilities but also reduced Aβ plaque deposition and inhibited tau protein hyperphosphorylation, thereby alleviating neuronal synaptic damage [79].

**Figure 4:**
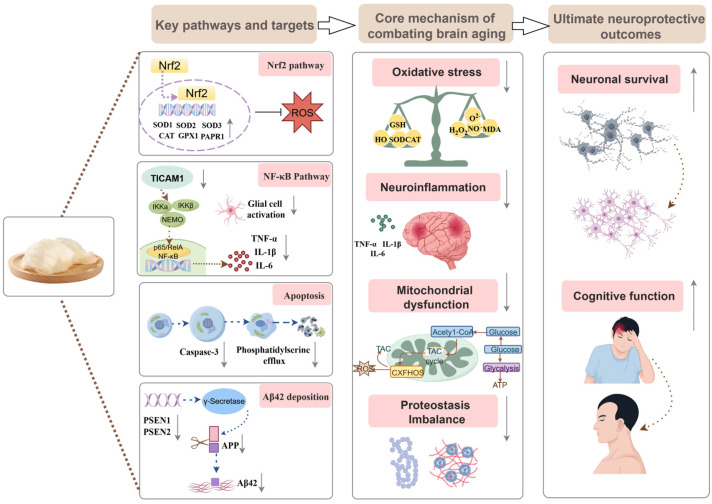
Integrative network-regulatory model of edible bird’s nest (EBN) against brain aging. This diagram is divided into two main sections. Key molecular pathways and targets regulated by EBN include: the Nrf2 antioxidant pathway (downstream targets SOD1-3, CAT, GPX1, PARP1), the TICAM1/NF-κB neuroinflammatory pathway (triggering glial cell activation and pro-inflammatory cytokines TNF-α, IL-1β, IL-6), apoptosis markers (Caspase-3, oxidative stress markers), and amyloid-related genes associated with Aβ42 deposition (PSEN1, PSEN2, APP). The core pathological mechanisms of brain aging counteracted by EBN encompass oxidative stress, neuroinflammation, mitochondrial dysfunction, and the interplay among these processes. Colored boxes represent distinct pathway categories or pathological mechanisms. Arrows indicate activation or inhibition, while lines with horizontal bars denote inhibitory effects. All abbreviations are defined in the text. This figure was created using Figdraw (Version 2.0, www.figdraw.com).

To better conceptualize the multi-level effects of EBN, we summarize the major molecular targets and functional outcomes in an integrative schematic (Figure 4). The figure illustrates how EBN modulates antioxidant, anti-inflammatory, apoptotic, mitochondrial, and proteostatic pathways, which together influence core mechanisms of brain aging and contribute to neuronal protection and cognitive support.

To date, there is a near-complete absence of high-quality RCTs evaluating EBN’s impact on cognitive function or progression to neurodegenerative diseases in human populations. The current preclinical literature collectively indicates that EBN exerts neuroprotective effects at multiple biological levels in both cellular and animal models. Nevertheless, the strength and translational significance of this evidence should be interpreted with appropriate caution. In several animal studies, the observed behavioral improvements appear not only statistically significant but also biologically meaningful. For instance, in the ovariectomized mouse model, high-dose EBN reversed memory deficits and, in some tasks, achieved effects comparable to those of estrogen replacement therapy. This suggests that the reported outcomes are not merely marginal changes but may reflect functionally relevant recovery. At the same time, most studies rely on group-wise comparisons without standardized reporting of effect sizes, which limits quantitative comparison across experiments. Another issue concerns the heterogeneity of experimental materials. EBN preparations differ substantially among studies in terms of species origin, geographic source, processing, and extraction methods. Although commonly used disease models, such as LPS induced neuroinflammation and 6-OHDA induced dopaminergic injury, are well established, variability in EBN preparation complicates the interpretation of whether differences in efficacy arise from biological mechanisms or methodological factors. Most current studies also employ relatively small sample sizes and acute or accelerated pathological paradigms. These models are valuable for mechanistic exploration, but they do not fully recapitulate the slow and progressive nature of human brain aging. Consequently, the reported effects should be viewed primarily as evidence of mechanistic plausibility rather than direct indicators of clinical benefit. Taken together, the existing preclinical data provide a solid biological rationale for EBN’s neuroprotective potential, but they mainly serve as a foundation for carefully designed human studies rather than definitive proof of efficacy.

Although direct experimental evidence is currently insufficient to demonstrate that EBN directly delays brain aging via the MGBA, its unique composition and observed physiological effects suggest considerable potential for exerting neuroprotective effects through modulation of this axis. Research findings suggest that EBN ameliorates gut microbiota dysbiosis by inhibiting the growth of pathogenic bacteria, promoting the proliferation of beneficial bacteria, and stimulating the production of SCFAs. In comparison to monosaccharide SA, EBN demonstrates superior prebiotic activity [80]. Further studies have shown that prolonged consumption of EBN enhances the abundance of beneficial gut bacteria such as *Bacteroides* and *Akkermansia*, while concurrently reducing the prevalence of Firmicutes. This suggests that EBN consumption may regulate gut function by promoting beneficial microbial communities. Moreover, EBN-derived gut metabolites can directly stimulate the proliferation of intestinal epithelial cells, thereby reinforcing the physical barrier of the intestinal wall [81]. In conclusion, current research tentatively supports the notion that EBN exhibits prebiotic-like properties by modulating the gut microbiota. The specific mechanisms involved include the promotion of beneficial bacteria, the inhibition of pathogenic bacterial growth, and the optimization of microbial community structure. Simultaneously, EBN enhances intestinal barrier function by stimulating the production of SCFAs and directly promoting the proliferation of intestinal epithelial cells. These effects provide essential theoretical support for the neuroprotective effects of EBN via the MGBA. While direct evidence concerning its role in mitigating brain aging requires further exploration, EBN shows considerable research potential and promising application prospects.

## 5. Comparison of EBN with Other Anti-Cognitive Decline Substances

The fundamental pathological mechanisms underlying brain aging encompass a multitude of pathways, with oxidative stress receiving considerable attention as a pivotal link among various mechanistic hypotheses. This section undertakes a comparative analysis of EBN with antioxidants and other dietary substances that counteract brain aging, aiming to elucidate its distinctive properties and identify existing research gaps. This analysis serves as a reference for the development of future intervention strategies.

### 5.1. EBN and Traditional Antioxidants

Antioxidants are classified into endogenous and exogenous types. Endogenous antioxidants, synthesized within the body, are further divided into enzymatic and non-enzymatic categories. In contrast, exogenous antioxidants are acquired through dietary sources [82], including vitamin E, vitamin C (ascorbic acid), and coenzyme Q10.

#### 5.1.1. Vitamin E

Vitamin E (VE) encompasses a group of fat-soluble compounds characterized by significant antioxidant properties. Structurally, VE is composed of tocopherols and tocotrienols, each of which exists in four isomeric forms: α, β, γ, and δ [83]. Notably, approximately 90% of VE present in plasma and brain tissue samples is in the form of α-tocopherol [84]. Research suggests that VE plays a crucial role in maintaining cellular redox homeostasis by neutralizing reactive ROS and mitigating lipid peroxidation. Supplementation with α-tocopherol has been shown to enhance cognitive function in patients with dementia, reduce the risk of cognitive decline, and stabilize cognitive performance [85]. Nevertheless, some studies report no significant benefits in certain subpopulations with specific dietary backgrounds, and excessive or high-dose supplementation may disrupt the equilibrium of endogenous antioxidant enzyme systems [83,86]. Unlike EBN, VE is capable of integrating into lipid structures within the central nervous system, where it acts synergistically with other intracellular antioxidants to inhibit lipid peroxidation induced by oxygen radicals [87].

#### 5.1.2. Vitamin C

Vitamin C (VC), also referred to as ascorbic acid, is a water-soluble antioxidant that mitigates oxidative damage by neutralizing oxygen-free radicals [88]. A deficiency in VC can lead to increased production of ROS and heightened oxidative stress through mitochondrial respiration, thereby contributing to the pathogenesis of AD [89]. In addition to its inherent antioxidant capabilities, VC alleviates oxidative stress by regenerating oxidized VE and enhancing the reductive capacity of NADH and NADPH [90]. Empirical studies have shown that individuals who supplement with VC experience a 16% reduction in the risk of developing AD compared to control groups [91]. Nevertheless, under certain redox conditions, VC may exhibit pro-oxidant effects, which are dependent on the individual’s redox status, dosage, and the route of administration [92]. Conversely, EBN has shown no significant toxicity in animal studies, although its long-term safety in humans necessitates further investigation.

#### 5.1.3. Coenzyme Q10

Coenzyme Q10 (CoQ-10) is an endogenous, lipophilic antioxidant that is highly concentrated in mitochondria, where it plays a pivotal role in cellular energy metabolism and antioxidant defense mechanisms. It exists in two primary forms: the oxidized coenzyme Q10 (ubiquinone) and its reduced counterpart (ubiquinol) [93]. Within the mitochondria, CoQ-10 functions as a critical electron carrier in the mitochondrial electron transport chain during oxidative phosphorylation, facilitating electron transfer from Complexes I and II to Complex III. Additionally, it is involved in the metabolism of pyrimidines and fatty acids, and acts as a cofactor for dihydroorate dehydrogenase in mitochondrial uncoupling proteins, as well as in the regulation of the mitochondrial permeability transition pore [94]. Beyond its mitochondrial functions, CoQ-10 is also localized in other subcellular organelles, including lysosomes, peroxisomes, the Golgi apparatus, and the endoplasmic reticulum. It is ubiquitously distributed throughout the brain, present in both neurons and glial cells [95]. Research has demonstrated that dietary supplementation with CoQ-10 ameliorates the pathological phenotype of the brain and enhances cognitive function in transgenic mouse models of AD. Mechanistically, CoQ-10 administration significantly reduced oxidative stress levels in the brain, evidenced by decreased carbonyl density and decreased Aβ deposition [96]. Similarly, in rat models of AD, intracerebroventricular injection of Aβ resulted in pronounced oxidative stress, indicated by increased serum malondialdehyde (MDA, a terminal product of lipid peroxidation) and elevated total oxidant levels. Further investigations have shown that CoQ-10 supplementation not only effectively mitigated this oxidative damage but also significantly enhanced the organism’s overall antioxidant capacity [97].

In conclusion, while traditional antioxidants primarily function as scavengers, brain aging is a multifaceted process involving various pathways, including oxidative stress, inflammation, and apoptosis. EBN, through the synergistic action of its multiple components, exhibits broader potential in combating brain aging by modulating the body’s antioxidant defenses, controlling inflammation, and supporting neurotrophic systems.

### 5.2. EBN and Other Food-Derived Sources with Anti-Brain-Aging Properties

With the progression of nutritional neuroscience, the identification of substances with neuroprotective properties in everyday diets has emerged as a central focus in research aimed at combating brain aging. These compounds exert their influence on brain health through various bioactive mechanisms, including antioxidant effects, anti-inflammatory properties, and neurotransmitter regulation. To further contextualize EBN’s potential, we compare it with other dietary strategies that have attracted substantial research interest in brain health. The following discussion contrasts EBN’s multi-component, food-matrix approach with interventions centered on isolated, high-potency phytochemicals (e.g., curcumin, quercetin) or essential nutrients (e.g., omega-3 fatty acids). Specifically, curcumin and quercetin are included as representative plant-derived polyphenols with potent anti-inflammatory and antioxidant properties [98,99], omega-3 fatty acids as essential nutrients crucial for neuronal membrane integrity and synaptic function [100], and lutein as a major carotenoid with antioxidant and neuroprotective effects [101]. In addition to single-entity compounds, we further include medicinal mushrooms (e.g., *Hericium erinaceus* and *Ganoderma lucidum*) and ginseng (*Panax ginseng*) as representative traditional multi-component functional foods. These categories are characterized by complex phytochemical or fungal metabolite matrices that act through coordinated modulation of neurotrophic, anti-inflammatory, and antioxidant pathways rather than a single dominant molecule [102,103].

This structured comparison aims to contrast EBN’s multi-component, food-matrix approach with the paradigm of high-potency, single-entity compounds and essential nutrients. This comparison is intended to illustrate different paradigms of nutritional intervention rather than direct equivalence in composition.

#### 5.2.1. Representative Polyphenol: Curcumin

Curcumin, chemically known as 1,7-bis (4-hydroxy-3-methoxyphenyl)-1,6-heptadiene-3,5-dione, is the principal polyphenolic component of turmeric and is characterized by its high lipophilicity [104]. The neuroprotective effects of curcumin on the aging brain are mediated through multiple signaling pathways, including the reduction in oxidative stress, inhibition of Aβ formation, and attenuation of neuroinflammation. Inhibition of NF-κB reduces inflammatory responses, while activation of nuclear factor erythroid 2-related factor 2 (Nrf2) promotes the expression of antioxidant response elements. Additionally, curcumin enhances autophagy and the expression of neurotrophic factors, thereby facilitating the clearance of deleterious protein aggregates [105]. In aged female Wistar rats, intraperitoneal administration of curcumin at 30 mg/kg (5 days weekly for 8 weeks) suggests potential in delaying brain aging by reducing oxidative stress, inhibiting apoptosis, and enhancing SOD activity, which in turn increases vascular endothelial growth factor (VEGF) and brain-derived neurotrophic factor (BDNF) expression [106]. Curcumin also appears to improve cognitive function by decreasing GSK-3β activity through the PI3K/Akt pathway [107]. In cellular models, it mitigates oxidative damage in aged PC12 cells, reduces reactive oxygen species production, prevents mitochondrial membrane potential loss, and promotes Nrf2 nuclear translocation to maintain mitochondrial function [108]. Preliminary clinical studies suggest curcumin supplementation may aid memory consolidation, enhance cognitive function, and reduce age-related memory decline [109]. Meta-analyses reveal that curcumin supplementation, particularly at 0.8 g/day for at least 24 weeks, effectively enhances cognitive function, with more significant benefits observed in elderly and Asian populations compared to younger and Western groups [110]. Curcumin is characterized by limited intestinal absorption and rapid hepatic metabolism, leading to extremely low bioavailability, which necessitates enhancement through nanocarriers [111]. Feng et al. demonstrated that the intranasal administration of curcumin molecules, which self-assemble into carrier-free curcumin nanoparticles, can inhibit the TLR4/NF-κB pathway that mediates the polarization of glial cells from the M1 to the M2 phenotype. This process reduces the Aβ burden in AD transgenic mice and exhibits stronger inhibitory effects on Aβ aggregation compared to free curcumin with monovalent effects [112].

#### 5.2.2. Representative Lipid: Deep-Sea Fish Oil (Omega-3 Fatty Acids)

Omega-3 fatty acids, particularly docosahexaenoic acid (DHA) and eicosapentaenoic acid (EPA), are critical components of fish oil. They play an essential role in maintaining neuronal membrane stability, synaptic plasticity, and anti-inflammatory signaling pathways, thereby supporting brain health and delaying cognitive decline [113]. Brain tissue contains a high concentration of DHA, accounting for 40% of synaptic membrane lipids. In contrast, EPA levels in the brain are 10 to 20 times lower, yet EPA remains crucial for microglial function [114]. Research has demonstrated a correlation between the intake of EPA and DHA and enhanced cognitive health in later life, as well as a reduced risk of dementia over a lifetime [115]. Several mechanisms have been proposed to explain these effects, including the enhancement of neurogenesis and the modulation of inflammation and neuroinflammation through the production of bioactive lipid mediators. A recent prospective study involving older adults examined the relationship between fish oil supplementation, which is rich in EPA and DHA, and the risk of developing dementia. The study found that over an average follow-up period of 7.92 years, 2054 participants were diagnosed with dementia. After adjusting for major risk factors, the intake of fish oil supplements was significantly associated with a reduced risk of dementia in adults aged 60–73 years (Odds Ratio = 0.87, 95% Confidence Interval: 0.79–0.96, *p* = 0.004) [116]. However, results from clinical trials have been inconsistent and varied, likely due to significant heterogeneity in trial designs [117]. Additionally, DHA has been shown to promote synapse formation by increasing the expression of drebrin, a dendritic spine marker protein, and PSD-95, a postsynaptic density protein [118]. DHA and EPA facilitate the dissociation of Nrf2 from its inhibitor Keap1, and promote the translocation of Nrf2 to the cell nucleus, where it interacts with the antioxidant response element [119]. Additionally, research indicates that EPA competes with arachidonic acid (ARA) for binding to cyclooxygenase (COX) and 5-lipoxygenase (5-LOX) enzymes, thereby attenuating ARA metabolism and decreasing the production of ARA-derived inflammatory mediators. DHA also competes with ARA for incorporation into cell membranes, thereby reducing neuroinflammation [120]. In contrast to EBN, DHA and EPA serve as essential ‘building blocks’ for the nervous system, whereas EBN primarily functions as a ‘regulatory modulator’ without providing foundational structural components.

#### 5.2.3. Representative Carotenoid: Lutein

Carotenoids, naturally occurring pigments prevalent in fruits and vegetables, are categorized into lipophilic carotenes, such as lycopene, α-carotene, and β-carotene, and more polar xanthophylls, including lutein, zeaxanthin, astaxanthin and β-cryptoxanthin. Lutein is notably the predominant carotenoid in the brain [121]. Its neuroprotective properties are attributed to its antioxidant activity, its ability to prevent neuroinflammation, and its role in enhancing levels of GAP-43, neural cell adhesion molecule (NCAM), and BDNF, which collectively promote neurogenesis and improve neural plasticity [122]. As an effective free radical scavenger, lutein demonstrates a particularly high capacity for quenching singlet oxygen, a capability directly linked to its extensive conjugated double bond structure [121]. Research conducted by Leila Nazari et al. has shown that lutein significantly alleviates cognitive impairment in a rat model of AD induced by Aβ [123]. Various behavioral assessments, including the Morris water maze and novel object recognition tests, have confirmed that lutein intervention effectively reduces deficits in spatial memory and recognition in these model animals. At the mechanistic level, lutein significantly ameliorated abnormal oxidative stress levels in the serum of AD rats. This was evidenced by a reduction in MDA content and total oxidation status, coupled with an increase in total antioxidant capacity. Similarly, another study demonstrated that intraperitoneal administration of lutein enhanced mitochondrial function by activating the Nrf2 pathway, thereby attenuating inflammation in C57BL/6J mice subjected to traumatic brain injury [124]. In a H_2_O_2_ induced oxidative stress model of BV-2 microglia, lutein treatment significantly upregulated the synthesis of SOD2, suggesting that lutein mitigates oxidative damage by augmenting endogenous antioxidant defenses [125]. Moreover, lutein has been shown to upregulate SIRT1 expression by inhibiting miR-135b-5p, which in turn suppresses M1 glial cell polarization and inflammation, thereby ameliorating PD [126]. However, the bioavailability of lutein is limited due to its intrinsic physicochemical properties. Its pronounced hydrophobicity hinders dissolution and transport within the aqueous environment of the gastrointestinal tract, thereby obstructing efficient delivery to absorption sites in the small intestine. Additionally, the conjugated double bond system within its molecular structure makes it prone to oxidative degradation during processing and storage, thereby compromising its stability and efficacy [127]. Similar to curcumin, lutein requires the use of nano-carriers to enhance its therapeutic efficacy.

#### 5.2.4. Representative Flavonoid: Quercetin

Flavonoids, a class of polyphenolic compounds prevalent in plant-based foods such as fruits, vegetables, tea leaves, and wine, exhibit significant neuroprotective potential across various subclasses. Notably, flavanols such as quercetin demonstrate substantial efficacy in inhibiting Aβ aggregation and mitigating AD-associated pathology [128]. Quercetin (3,3′,4′,5,7-dihydroxyflavone) is abundantly found in plants, including apples, onions, broccoli, wine, green tea, and *Ginkgo biloba* [129]. In addition to directly targeting Aβ, quercetin reduces oxidative stress, inhibits neuroinflammation, enhances mitochondrial function, and promotes neuronal survival [128]. Research suggests that quercetin can traverse the BBB and exert neuroprotective effects by modulating multiple signaling pathways, including the Nrf2/ARE pathway, which serves as a fundamental defense mechanism for cells against oxidative damage [130]. Research has demonstrated that quercetin intervention at a dosage of 60 mg/kg/day effectively reverses cognitive impairment in AD model mice and significantly suppresses the expression of pro-inflammatory cytokines, including TNF-α, IL-6, and IL-1β, within the brain. The study further elucidated that the neuroprotective effects of quercetin are closely linked to its activation of the Nrf2/HO-1 signaling pathway and its facilitation of M2 anti-inflammatory phenotype polarization in microglia [131]. Moreover, evidence suggests that quercetin modulates multiple downstream pathways, such as PI3K/Akt signaling activation, CREB phosphorylation, and NF-κB inhibition, by upregulating the expression of key neurotrophic factors like BDNF and nerve growth factor (NGF). These synergistic effects collectively enhance neuronal resilience, thereby promoting improvements in memory and learning functions [132]. However, it is important to note that due to its poor water solubility and low BBB permeability, quercetin exhibits an oral bioavailability of approximately 2% [133]. To address this challenge, numerous researchers have utilized nano-delivery systems and liposomal surface modifications to improve the efficiency of BBB penetration. A multifunctional nanoparticle platform, designated as Anti-VCAM1-GM1@Q, has been developed. This platform targets vascular cell adhesion molecule 1 on brain vascular endothelial cells, inhibiting its signaling pathway, while concurrently employing monosialoglycerolipid to enhance the ability of quercetin to traverse the BBB. This dual mechanism exerts synergistic anti-inflammatory effects, significantly mitigating neuroinflammation and cognitive impairment [134]. Furthermore, the menthol-modified quercetin liposomes (Men-Qu-Lips) developed by Liu et al. exhibited exceptional encapsulation efficiency and stability. These liposomes effectively crossed the BBB, reducing oxidative stress and neuroinflammation in the brains of aging mice, thereby protecting neurons and enhancing their learning and memory capabilities [133].

#### 5.2.5. Representative Functional Products: Medicinal Mushrooms

Medicinal mushrooms, including *Hericium erinaceus* (lion’s mane) and *Ganoderma lucidum* (reishi), represent a class of food-derived complex natural products with growing evidence for neuroprotective and anti-Alzheimer’s potential. Lion’s mane mushroom has been widely studied for its rich profile of bioactive compounds such as erinacines and hericenones, which have been shown to stimulate the synthesis of NGF and support neurite outgrowth, thereby contributing to neuronal maintenance and cognitive function in vitro and in vivo models of neural injury and neurodegenerative pathology (e.g., NGF promotion and neurite enhancement) [135]. Beyond its neurotrophic effects, Lion’s mane mushroom extract exhibits antioxidant and anti-inflammatory properties. It helps mitigate oxidative stress and inflammation by regulating cellular defense pathways and cytokine production [102]. Preclinical studies have also demonstrated that lion’s mane polysaccharide-enriched extracts reduce ROS accumulation, prevent mitochondrial depolarization, and preserve cholinergic system function in AD models, further highlighting their multi-mechanistic neuroprotective capacities [136]. Moreover, emerging review evidence supports the idea that *H. erinaceus* bioactive constituents act synergistically to regulate neurotrophic, antioxidative, and anti-cytotoxic processes, positioning this mushroom as a functional dietary candidate for cognitive support and age-related neuronal health [137].

Reishi mushroom (*G. lucidum*) and other medicinal fungi exhibit complementary neuroprotective potential through distinct classes of bioactive metabolites, including triterpenoids and polysaccharides, which have been shown to modulate neuroinflammation via pathways such as TLR4/NF-κB and attenuate microglial overactivation associated with neurodegenerative progression [103]. Systematic analyses suggest that multiple medicinal mushroom species (e.g., *Hericium*, *Ganoderma*, *Cordyceps*) may alleviate cognitive impairment in animal models, indicating that mushroom supplementation could contribute to neuronal resilience through diverse mechanisms, including antioxidation, immunomodulation, and regulation of neurotrophic signaling [138]. While clinical evidence remains limited, the cumulative preclinical data provide a strong rationale for further investigation of medicinal mushroom-derived compounds as functional food strategies for supporting cognitive function and mitigating neurodegenerative risk.

#### 5.2.6. Representative Traditional Multi-Component: Ginseng

Ginseng has been widely studied for its neuroprotective potential in the context of AD, with multiple major components, including ginsenosides and gintonin, demonstrating modulation of key pathological processes such as Aβ metabolism, neuroinflammation, oxidative stress, and mitochondrial dysfunction [139]. Ginsenosides, the principal bioactive saponins in *Panax ginseng*, have been reported to target multiple mechanisms in AD models, including attenuation of Aβ formation, inhibition of tau hyperphosphorylation, and enhancement of neurotrophic signaling [139]. In preclinical studies, isolated ginsenosides (e.g., Rg1) have been shown to improve cognitive performance and reduce amyloid plaque burden in animal models by activating antioxidant and anti-inflammatory pathways, as well as supporting cholinergic system function [140,141]. Mechanistically, ginsenosides exhibit anti-neuroinflammatory effects through inhibition of pro-inflammatory cytokine production and modulation of NF-κB related signaling, which are implicated in AD pathology [140]. Furthermore, several clinical and observational studies suggest that long-term ginseng consumption is associated with cognitive benefits in the elderly, pointing to its potential utility as a preventive dietary strategy for age-related cognitive decline, although findings are still preliminary and warrant further large-scale trials [142].

**Figure 5:**
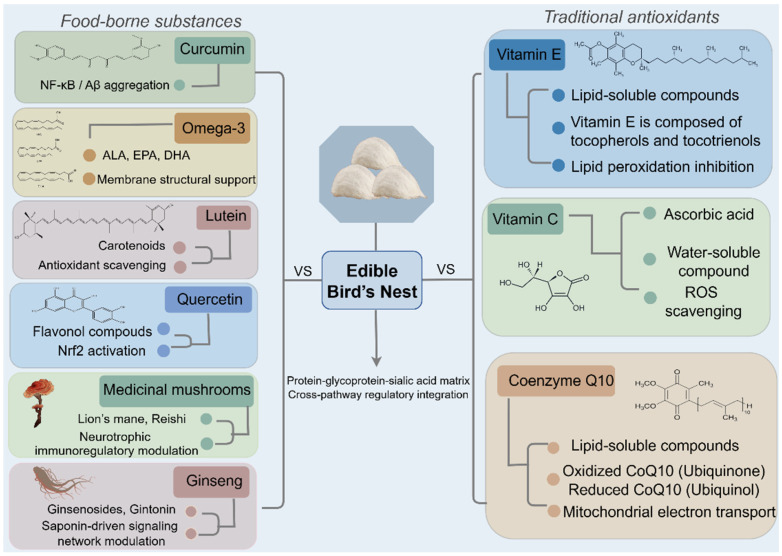
Comparison of edible bird’s nest with food-borne substances and traditional antioxidants. This diagram is divided into three major categories: food-derived substances (curcumin, omega-3 fatty acids, lutein, quercetin) and their key molecular targets and mechanisms of action; traditional antioxidants (vitamin E, vitamin C, coenzyme Q10) and their primary functions; and EBN, positioned as a multi-component protein-glycoprotein-sialic acid matrix with cross-pathway regulatory integration capabilities. Abbreviations: ALA, α-linolenic acid; EPA, eicosapentaenoic acid; DHA, docosahexaenoic acid; ROS, reactive oxygen species; Nrf2, nuclear factor erythroid 2-related factor 2; NF-κB, nuclear factor kappa-light-chain-enhancer of activated B cells. This figure was created using Figdraw (Version 2.0, www.figdraw.com).

Through a systematic comparison of various food-derived anti-brain aging compounds, it becomes apparent that each compound offers distinct benefits and limitations in addressing cognitive decline, attributable to their unique chemical properties and mechanisms of action. Curcumin and quercetin feature broad signaling interactions but are constrained by bioavailability; omega-3 fatty acids serve as structural components; lutein targets oxidative stress and synaptic pathways; medicinal mushrooms and ginseng provide multi-component bioactivity but from different biochemical matrices. In comparison, EBN’s value lies in its multi-target network-regulatory capacity, engaging antioxidant, anti-inflammatory, apoptotic, and neurotrophic pathways within a single food matrix. This integrated regulatory pattern potentially bridges the gap between traditional antioxidant supplementation and structurally oriented nutrient interventions, supporting cognitive resilience in aging (Figure 5).

## 6. Safety, Quality Control, and Regulatory Considerations of EBN

In addition to efficacy, the translational development of EBN as a functional food or nutraceutical necessitates rigorous attention to safety, quality control, and regulatory compliance. IgE-mediated allergenicity has been reported in sensitive individuals, particularly in children, indicating that EBN proteins can act as food allergens and should be clearly labeled for consumer safety [21]. Furthermore, the composition of EBN varies considerably depending on swiftlet species, geographic origin, nesting environment, and post-harvest processing methods, leading to substantial batch-to-batch variability in bioactive profiles [16,143]. Such heterogeneity presents challenges for dose standardization and reproducibility of biological effects. Contamination issues also warrant attention. Several studies have reported the presence of heavy metals such as Pb, Hg, and Cd in EBN samples, likely derived from environmental exposure and processing conditions [36]. These findings highlight the necessity of rigorous quality control systems, including raw material screening, standardized processing, and contaminant monitoring. From a regulatory perspective, EBN is generally classified as a traditional food in many Asian countries, whereas in Western contexts it may fall under the category of nutraceuticals or novel foods. This distinction has important implications for safety evaluation, labeling, and health-claim substantiation. Future translational research should therefore integrate toxicological assessment, dose optimization, and regulatory alignment to ensure the safe and effective use of EBN in brain health applications.

## 7. Future Research Directions

In mechanistic studies, it is essential to establish humanized in vitro models of the BBB, such as co-culture systems comprising hCMEC/D3 cells and astrocytes, to assess the penetration efficiency and transport mechanisms of active components in EBN. This approach will facilitate the validation of EBN’s impact on BBB permeability. In clinical research, there remains a pronounced lack of RCTs and long-term follow-up data in middle-aged and elderly populations at risk for neurodegenerative disorders. Future studies should therefore prioritize rigorously designed RCTs. Importantly, the sample size for definitive trials should not be arbitrarily defined but determined through a priori power calculations based on effect sizes and variance estimates obtained from well-designed pilot studies. Trial design should include clearly defined and clinically relevant primary cognitive endpoints, such as changes in composite scores derived from validated batteries (e.g., ADAS-Cog or MoCA), together with secondary outcomes reflecting daily functioning and neuropsychiatric status. In addition, stratification or adjustment for key confounders, including sex, baseline dietary habits (including prior exposure to EBN), and genetic or metabolic risk factors, will be essential to ensure interpretability and generalizability of findings. For industrial applications, the development of co-encapsulated EBN-probiotic microcapsules is recommended to achieve sustained intestinal release and synergistic enhancement of the microbiota.

## 8. Conclusions and Outlook

In conclusion, EBN exhibits promising potential for application in mitigating brain aging. Its antioxidant, anti-inflammatory, and neuroprotective properties may contribute positively to decelerating the brain aging process. Active constituents such as SA, glycoproteins, and EGF are postulated to exert advantageous effects on nervous system function. Nonetheless, rigorous basic experimental and clinical research is imperative to scientifically substantiate its neuroprotective mechanisms and effects. Future investigations should focus on elucidating the molecular and cellular mechanisms of EBN, thereby providing a theoretical basis for the development of anti-brain-aging therapeutics. Additionally, its potential utility in the prevention and treatment of neurodegenerative diseases warrants evaluation to improve the quality of life for the elderly population.

## Figures and Tables

**Table 1:** Animal and cell studies on the effects of EBN and SA intervention in brain aging.

Model	Species	Tested Material and Dosage	Duration	Experimental Results	Ref.
Menopause-related cognitive decline (Ovariectomy)	SD rat	EBN mixed with diet at concentrations of 6%, 3%, and 1.5% (*w*/*w*)	12 weeks	Reduced escape latency time Increased expression of SIRT1 in the hippocampus Reduced levels of caspase-3 and malondialdehyde in the hippocampal region and frontal cortex Reduced PSEN1, PSEN2 and APP gene expression Increased levels of SOD1, SOD2, SOD3, and CAT	[70,71]
Neuroinflammation (LPS)	Wister rat	EBN water extract of 125, 250 and 500 mg/kg	7 days	Reduced escape latency time Reduced levels of TNF-α, IL-1β, IL-6, ROS and TBARS	[72]
Neuroinflammation (LPS)	C57BL/6J mice	EBN of 200 mg/kg/d	7 weeks	Reduced escape latency time Increased neuronal count Inhibition of glial cell activation Reduced levels of TNF-α, IL-1β, and IL-6 Inhibited expression of TICAM1	[73]
PD (6-OHDA)	C57BL/6J mice	Pancreatic enzyme-digested extract and aqueous extract of EBN at 20 mg/kg and 100 mg/kg, respectively.	28 days	Improved locomotor distance and balance Reduced loss of TH-positive neurons in the substantia nigra Decreased expression of the microglial marker CD11b Increased expression of the antioxidant marker GPX1	[74]
AD (2VO)	SD rat	EBN of 60 and 120 mg/kg	8 weeks	Increased neuronal count in the hippocampal CA1 region Reduced hippocampal F2-prostaglandin levels	[75]
Vascular dementia (MCAO)	Wister rat	EBN water extract powder of 10, 50 and 100 mg/kg	2 weeks before and 3 weeks after surgery	Reduced escape latency time Decreased Aβ42 deposition in the hippocampus Inhibited AChE activity	[17]
PD (6-OHDA)	SH-SY5Y cells	Pancreatic enzyme-digested extract and aqueous extract of EBN at 1/2 MNTD and MNTD, respectively	-	NO production and reduced lipid peroxidation	[74]
PD (6-OHDA)	SH-SY5Y cells	Pancreatic enzyme digestion of crude extracts (75 μg/mL) and aqueous extracts (150 μg/mL)	-	Pancreatic enzyme extract demonstrates superior efficacy in enhancing cellular viability The aqueous extract exhibits greater effectiveness in mitigating ROS accumulation, alleviating early apoptotic membrane phosphatidylserine efflux, and inhibiting caspase-3 cleavage	[76]
Oxidative stress (H_2_O_2_)	SH-SY5Y cells	EBN (1000 μg/mL) and its constituents lactoferrin (LF, 5 μg/mL) and ovoferritin (OVF, 10 μg/mL)	-	Enhances free radical scavenging activity and reduces ROS Upregulates the expression of SOD1, SOD2 and PARP1 genes	[77]
Neural stem cell model	NE-4C	Pancreatic enzyme-digested crude extract (S1; MNTD = 130 μg/mL) and the aqueous extract (S2; MNTD = 290 μg/mL)	-	Promoting cell proliferation, migration and neuronal differentiation	[22]
AD	2 × Tg-AD mice	SA of 17, 84 and 420 mg/kg	9 months	Reduced escape latency time Decreased Aβ and neurofibrillary tangles Increased number of Nissl bodies	[79]
Mitochondrial dysfunction	SH-SY5Y cells	SA extracts at concentrations of 20, 40, 60, 80 and 100 μg/mL in EBN	-	Cell viability peaked at 60 μg/mL The number of active mitochondria increased significantly by 195%	[78]

## Data Availability

No new data were created or analyzed in this study. Data sharing is not applicable to this article.

## Abbreviations

EBN: Edible bird’s nest; AD: Alzheimer’s disease; PD: Parkinson’s disease; Ca: Calcium; Fe: Iron; GPC: Chromatography; RP-HPLC: Reverse-phase high-performance liquid chromatography; AM Case: Acidic mammalian chitinase; LOXL3: Lysyl oxidase homologue 3; MUC5AC: Mucin-5AC; NUCB2: Nucleobindin-2; MUC5B: Mucin 5B; LOXL2: Lysyl oxidase-like protein 2; CHIA: Chitinase; OIH: Ovomucoid-like protein; CAB45: Calcium-binding protein 45; GO: Gene Ontology; SA: sialic acid; Neu5Ac or NANA: Acetylneuraminic acid; Neu5Gc: N-glycolylneuraminic acid; KDN: Diamino neuraminic acid; Neu: Neuraminic acid; P: Phosphorus; Na: Sodium; K: Potassium; I: Iodine; Mg: Magnesium; Cr: Chromium; Se: Selenium; Pb: Lead; Cd: Cadmium; As: Arsenic; Hg: Mercury; EGF: Epidermal growth factor; NSCs: Neural stem cells; TPC: Total phenolic content; MCI: Mild cognitive impairment; ROS: Reactive oxygen species; NO: Nitric oxide; iNOS: Nitric oxide synthase; IL-17: Leukin-17; Aβ: β-amyloid; mt DNA: Mitochondrial DNA; TDP-43: Tar DNA-binding protein-43; SOD1: Superoxide dismutase 1; MGBA: Microbioa–gut–brain axis; BBB: Blood–brain barrier; SCFAs: Short-chain fatty acids; SIRT1: Sirtuin-1; LPS: Lipopolysaccharide; SOD: Superoxide dismutase; GPx: Glutathione peroxidase; AchE: Acetylcholinesterase; ACh: Acetylcholine; 6-OHDA: 6-hydroxydopamine; MNTD: maximum non-toxic dose; VE: Vitamin E; VC: Vitamin C; CoQ-10: Coenzyme Q10; VEGF: Vascular endothelial growth factor; BDNF: Brain-derived neurotrophic factor; DHA: Docosahexaenoic acid; EPA: Eicosapentaenoic acid; NCAM: Neural cell adhesion molecule; NGF: Nerve growth factor
